# Analysis of Allele-Specific Expression Highlights Novel Participants of Empagliflozin-Driven Effects on T2DM-Associated Regulatory Pathways

**DOI:** 10.3390/ijms27167202

**Published:** 2026-08-12

**Authors:** Elena E. Korbolina, Maria Gubina, Leonid O. Bryzgalov, Arina O. Degtyareva, Anastasia A. Evseenko, Elena V. Antonseva, Anton I. Korbut, Elena Y. Rykova, Vadim V. Klimontov, Julia G. Kzhyshkowska, Tatiana I. Merkulova

**Affiliations:** 1 Institute of Cytology and Genetics, Siberian Branch of Russian Academy of Sciences, 630090 Novosibirsk, Russiakorbutai@icgbio.ru (A.I.K.); rykova.elena.2014@gmail.com (E.Y.R.); klimontov@mail.ru (V.V.K.);; 2 VECTOR-BEST, P.O. Box 492, 630117 Novosibirsk, Russia; 3 Research Institute of Clinical and Experimental Lymphology—Branch of the Institute of Cytology and Genetics, Siberian Branch of Russian Academy of Sciences, 630090 Novosibirsk, Russia; 4 Laboratory of Translational Cellular and Molecular Biomedicine, National Research Tomsk State University, 634050 Tomsk, Russia; julia.kzhyshkowska@medma.uni-heidelberg.de; 5 Institute of Transfusion Medicine and Immunology, Institute for Innate Immunoscience (MI3), Medical Faculty Mannheim, University of Heidelberg, 68167 Mannheim, Germany

**Keywords:** type 2 diabetes mellitus, peripheral blood mononuclear cells, sodium–glucose cotransporter 2 inhibitor empagliflozin, response to therapy, RNA-seq, allele-specific expression, transcription factors, regulatory interactions

## Abstract

It is well-known that the morbidity and clinical burden of type 2 diabetes mellitus (T2DM) are predominantly associated with its chronic complications, in which fibrosis is a significant contributor. Recently, sodium–glucose cotransporter 2 (SGLT2) inhibitors have made a pivotal advancement in the therapeutic landscape not only improving glycemic control, but also demonstrating high effectiveness in the prevention and treatment of T2DM complications. In this work, we aimed to assess the transcription factors (TFs) mediating the effects of SGLT2 inhibitor empagliflozin (EMPA) treatment by a comprehensive analysis of the allele-specific expression (ASE) events utilizing the RNA-seq data. Initial logistic regression analysis of the in vitro transcriptomic data for EMPA-treated peripheral blood mononuclear cells (PBMCs) of three healthy donors revealed a significant inter-individual variation in ASE for 240 genes linked to EMPA treatment beyond the glucose-lowering effects. Then, 146 TFs were predicted to regulate the expression of the corresponding targets using motifbreakR and DESeq2. Among these, multiple TFs (including ATF3, ATF4, E2F1, EGR1, FOS, JUN, JUNB, IRF8, KLF6, KLF11, SNAI1, TWIST1, and ZEB1) were involved in the TGF-β/SMAD3 canonical profibrotic signaling cascade, pertinent to diabetes-related fibrosis, playing a significant role in the development of diabetic complications. Further analysis of the in vivo data for the PBMCs from ten T2DM patients initiating EMPA therapy identified 98 TFs related to the ASE variation in both in vitro and in vivo cohorts. To conclude, our integrative allele-specific approach enables the prediction of novel EMPA-responsive regulatory interactions and suggests the important mediators of the mechanisms underlying the effects of EMPA on human PBMCs.

## 1. Introduction

Diabetes mellitus (DM) is one of the main causes of mortality and severe complications, such as blindness, renal and/or cardiac failure, strokes, and lower limb amputation. According to the International Diabetes Federation (IDF), the total number of DM patients in the world as of 2024 was 589 million. The number of people living with DM is expected to increase to 853 million by 2050 [1]. Type 2 DM (T2DM) accounts for over 90–95% of all DM cases and is characterized by chronic hyperglycemia, developing as a result of a decrease in the tissue sensitivity to insulin (insulin resistance) and its impaired secretion [2,3]. Importantly, it is not the disease itself but rather its complications that account for most of the mortality, disability, and deterioration in quality of life associated with T2DM. These complications comprise chronic kidney disease, heart failure, hepatic insufficiency, retinopathy, and peripheral neuropathy [4,5,6,7]. That is why the current T2DM management strategy relies on individualization of the treatment taking into account the range of complications [8].

Sodium–glucose cotransporter 2 (SGLT2) inhibitors are a new group of antidiabetic drugs introduced into clinical practice after 2010; their popularity continuously increases to reach the current peak. SGLT2 inhibitors (SGLT2is) are the specific antagonists of SGLT2 transporters expressed in the proximal tubules of the kidneys. These inhibitors bind to the SGLT2 protein and inhibit its function, thereby blocking the glucose reabsorption from urine back to the bloodstream and thus reducing the blood glucose levels [9]. Since their introduction into clinical practice, SGLT2is have demonstrated their efficiency in prevention and treatment of T2DM cardiovascular and renal complications [9,10,11,12]. As has been shown, SGLT2is alleviate T2DM complications not only by their glucose-lowering effect; moreover, their beneficial effect often does not depend on it [13]. The results of EMPEROR, DAPA-HF, and DAPACKD trials demonstrate that empagliflozin and dapagliflozin decrease the risk of the heart and kidney damage in people both with and without T2DM [12,13,14,15].

The T2DM patients are subject to a significantly increased risk of the diabetic cardiomyopathy, heart failure, and myocardial infarction [13,16]. SGLT2is improve adverse cardiovascular events in T2DM through a combination of hemodynamic, metabolic, anti-inflammatory, antifibrotic, and vascular effects on the myocardium [16]. The data of numerous experimental studies demonstrated a beneficial action of SGLT2is on various cardiac cell types, such as cardiomyocytes, endothelial cells, fibroblasts, and smooth muscle cells [17]. These drugs reduce inflammation, oxidative stress, and apoptosis, as well as enhance endothelial function and energy utilization, thus improving the cardiac function [18]. Diabetic nephropathy is a severe microvascular DM complication with renal interstitial inflammation and fibrosis developed as a result of hyperglycemic conditions in T2DM. The mechanistic studies in animal models of diabetic kidney disease and cultured renal cells demonstrate the significant anti-inflammatory, antioxidative, and antifibrotic effects of SGLT2is [10,19,20]. A recent whole-transcriptome study of the kidney tissue [21] shows that empagliflozin (EMPA) improves the outcome of diabetic nephropathy in a mouse T2DM model by inhibiting inflammation, apoptosis, senescence, and other related pathways.

Despite considerable interest in the SGLT2is therapeutic potential, the mechanisms involved in their preventive effect against T2DM complications remain incompletely understood. Notably, one of the key mechanisms underlying the cardiovascular and renal protective effects of SGLT2is in T2DM patients is the reduction in chronic low-grade inflammation [19,22]. T2DM causes the development of this type of inflammation in the cardiovascular system, kidneys, adipose tissue, and other organs in which monocytes/macrophages play the leading role [19,23]. Numerous studies suggest the involvement of inflammatory macrophages (M1) in the development of diabetic complications [24,25,26,27]. Hyperglycemia is known to stimulate inflammatory reactions in monocytes/macrophages, inducing them to produce inflammatory cytokines and reactive oxygen species [28,29] exacerbating endothelial dysfunction in renal and heart complications of diabetes, as well as retinopathy [30]. The key signaling pathways that regulate macrophage polarization include NF-κB, JAK/STAT, and Notch [19]. As noted above, the suppression of the inflammatory programming of tissue-resident macrophages as well as recruited monocyte-derived macrophages by SGLT2is plays an important role in their anti-inflammatory effect [19,22].

The molecular mechanisms underlying the effects of SGLT2is on monocyte/macrophages remain to be elucidated. The most efficient way to gain insight into these mechanisms is provided by the state-of-the-art sequencing-based omics technologies, such as RNA-seq, scRNA-seq, ChIP-seq, DNase-seq, and ATAC-seq, able to provide the most comprehensive picture of the genes, regulatory pathways, and key transcription factors (TFs) involved in the development of T2DM and its complications, as well as the molecular mechanisms underlying the SGLT2is therapeutic and protective effects. In this study, we searched for the TFs that could mediate the effects of EMPA treatment by analyzing the allele-specific expression (ASE) events in the RNA-seq data obtained for the PBMCs of healthy donors treated with EMPA in vitro and the PBMCs from T2DM patients under EMPA therapy.

## 2. Results

### 2.1. Overview of the Computational Approach

**Figure 1:**
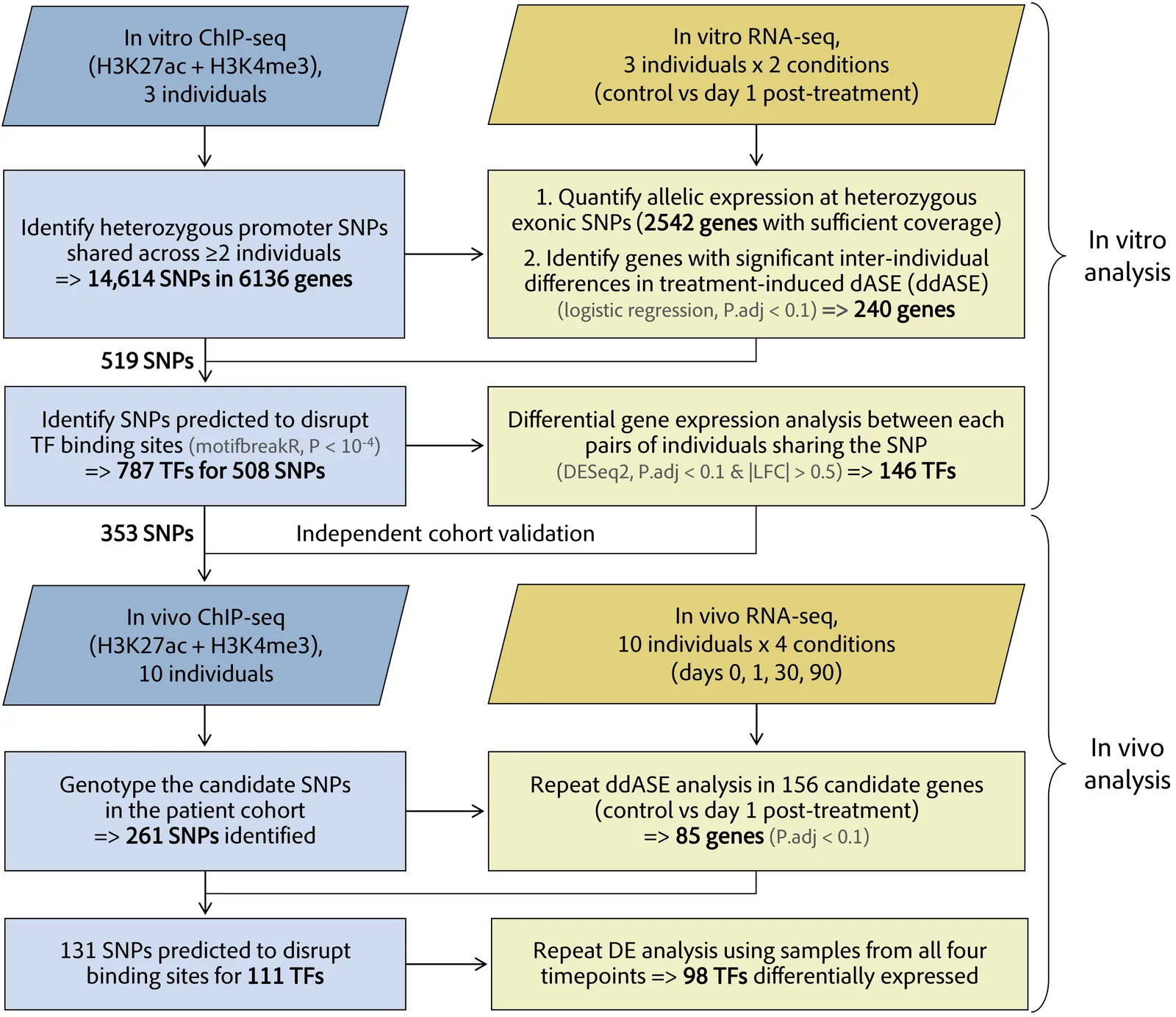
Integrative multi-omics workflow used to identify the transcription factors and the corresponding target genes associated with inter-individual variation in the transcriptional response to empagliflozin (EMPA). Abbreviations: TF, transcription factor; dASE, differential allele-specific expression; DE, differential expression.

Figure 1 shows the overall workflow aimed to identify novel TFs involved in gene expression control under EMPA treatment.

We hypothesized that the treatment-induced shifts in the relative abundance of allele-specific transcripts measured for heterozygous SNPs within the transcribed regions reflect allele-dependent differences in gene regulation. In this study, we attributed these regulatory effects to heterozygous SNPs predicted to alter the TF binding sites within promoter regions (regulatory SNPs, or rSNPs). The TFs predicted to bind these sites were considered candidate mediators of the EMPA response, particularly when the TF itself was differentially expressed between EMPA-treated and control samples.

The pipeline integrated two independent datasets:

(i) an in vitro dataset consisting of ChIP-seq (baseline condition) and RNA-seq (baseline and day 1 of EMPA-treatment) data generated from the EMPA-treated PBMCs sampled from three healthy donors;
(ii) an in vivo dataset from ten T2DM patients, including ChIP-seq data collected on day 0 and RNA-seq data collected on days 0, 1, 30, and 90 after the initiation of oral EMPA treatment.

To eliminate the confounding systemic glucose-lowering effects of EMPA and specifically focus on its immune-associated regulatory mechanisms, the initial analyses were performed using the in vitro PBMC dataset.

First, we identified the genes containing heterozygous promoter SNPs shared by at least two individuals using in vitro ChIP-seq data. The RNA-seq data from the same cohort were then used to identify the heterozygous SNPs within the transcribed regions of these genes and to quantify the allelic imbalance. Using these data, we applied logistic regression to identify the genes showing pronounced *inter-individual variability in differential allele-specific expression* (ddASE) in response to EMPA (see Section 4). This analysis identified the protein-coding genes that may be associated with the direct EMPA effects in PBMCs.

Then, based on our hypothesis, we applied motifbreakR [31] to assess whether the heterozygous SNPs in promoters of these genes can be considered regulatory SNPs (rSNPs), i.e., can affect gene expression by altering TF binding. For the resulting list of 787 TFs, pairwise differential expression analyses were performed using the in vitro RNA-seq data between the individuals carrying the same rSNP with DESeq2 (adjusted *p* < 0.1; |log2FC| > 0.5).

A list of 353 SNPs predicted to disrupt the sites of 146 differentially expressed TFs was further evaluated to determine whether the EMPA-associated effects were consistently reproducible in the cohort of T2DM patients (in vivo dataset). At this stage, we determined the individual genotypes based on the in vivo ChIP-seq data and identified the pairs of patients carrying the same heterozygous promoter variants for each gene. We then applied the same logistic regression framework to the in vivo RNA-seq dataset to identify the genes exhibiting a significant ddASE following the EMPA treatment. For TFs predicted to bind sites disrupted by promoter SNPs in these genes, we performed pairwise differential expression analyses between the corresponding patient pairs. For these analyses, RNA-seq data from all four available time points (days 0, 1, 30, and 90) were included in the DESeq2 model, thereby increasing statistical power for the detection of genotype-associated differences in TF expression.

This integrative approach enabled the identification of 98 TFs whose expression changes were consistently associated with the inter-individual variability in allele-specific expression differences in downstream target genes in both EMPA-treated primary PBMC cultures and PBMCs obtained from T2DM patients after EMPA administration.

In addition, we analyzed the correlation between the dASE of target genes and the change in expression of the corresponding TFs for 261 potential regulatory SNPs found among the T2DM patients. This analysis identified 16 genes for which treatment-induced dASE was associated with changes in the expression of the corresponding TFs, suggesting that these genes may represent their EMPA-responsive targets.

### 2.2. Determination of the EMPA Effects on Allele-Specific Expression In Vitro and the Search for Associated Transcription Factors

Genotyping based on the in vitro ChIP-seq data for histone marks identified 14,614 heterozygous SNPs occurring in at least two individuals and mapped to the promoter regions of 6136 genes. Using logistic regression analysis, we selected the genes that displayed the highest inter-individual variation in the response to EMPA at the level of differential allele-specific expression (ddASE) in the individuals sharing the same rSNPs. Each gene–patient pair was treated as an independent test. A total of 3680 comparisons involving 2542 distinct genes were analyzed. After Benjamini–Hochberg false discovery rate correction, 262 comparisons remained significant (adjusted *p* < 0.1), corresponding to 240 unique genes that could be functionally linked to the specific EMPA effects in PBMCs (Supplementary Table S1).

**Figure 2:**
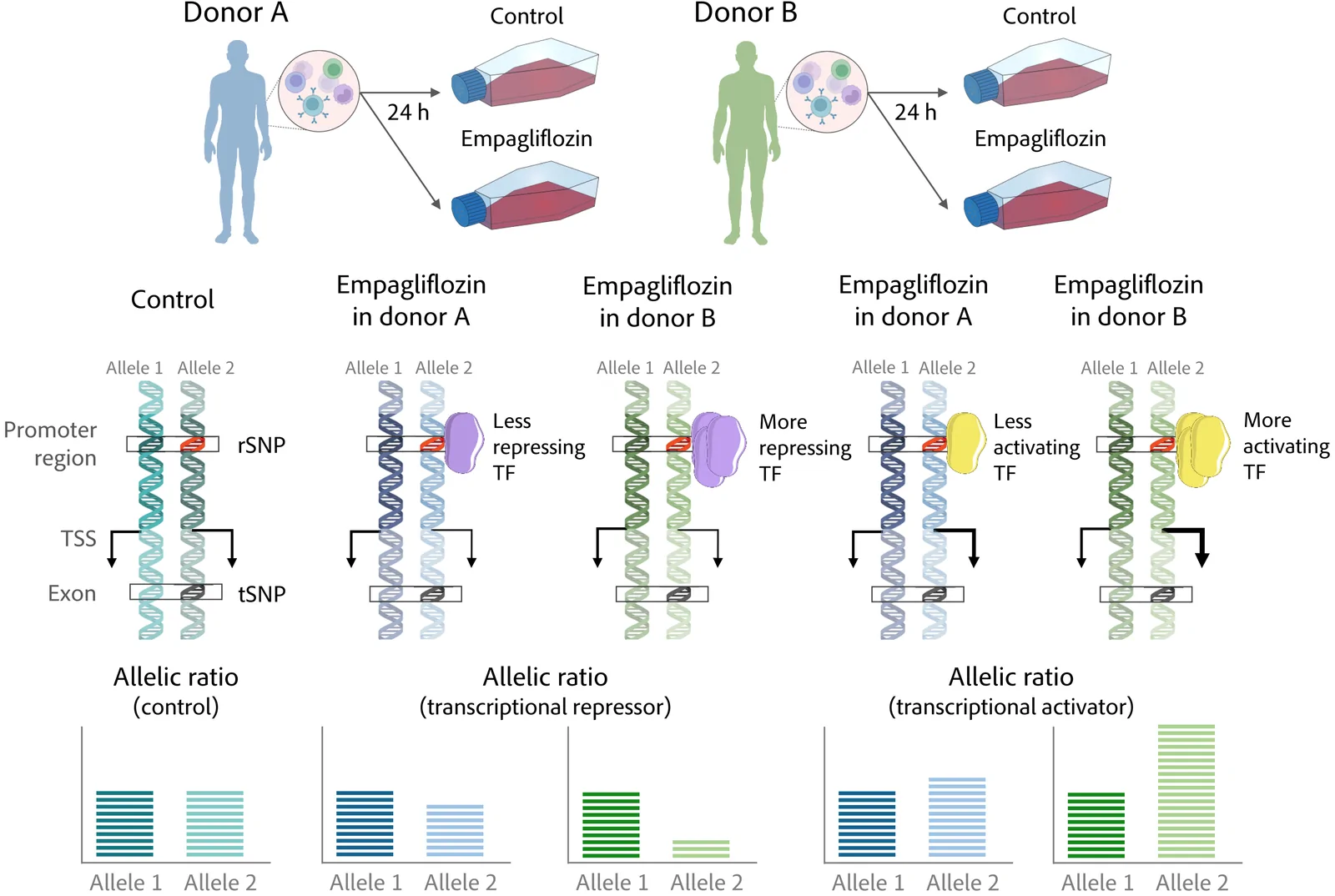
Proposed mechanism linking differential transcription factor abundance and inter-individual variation in differential allele-specific expression following empagliflozin treatment. Abbreviations: rSNP, regulatory SNP located in the promoter region of a gene exhibiting ddASE; tSNP, transcribed SNP located in an exon and used to assess allelic imbalance; TSS, transcription start site; and TF, transcription factor. The PBMCs sampled from different individuals are exposed to the same treatment; however, distinct regulatory landscapes lead to variable transcriptional responses between individuals. In the absence of regulatory effects, both alleles are considered to be expressed at comparable levels. A promoter-region regulatory SNP can alter the binding affinities in an allele-specific manner. Enhanced binding of an activating TF to one allele promotes preferential transcription from this allele, whereas preferential binding of a repressing TF suppresses transcription of the affected allele, resulting in allelic imbalance. Since the abundance of TFs differs between individuals, the magnitude of allele-specific imbalance may also vary between the subjects, thereby producing inter-individual differences in the treatment-induced dASE. Bar plots schematically represent the relative expression levels of two alleles in each regulatory scenario.

Our hypothesis implies that a significant difference in the dASE computed for these 240 genes is associated with the effect of a certain TF on gene expression (Figure 2), that is, the allele-specific regulatory effects were attributed to the rSNPs predicted to alter the binding sites of some TF/TFs within the gene promoter region. Differences in treatment-induced allele-specific regulatory effects between individuals carrying the same rSNP were hypothesized to result from differences in the expression of the corresponding TFs between two individuals.

Using the in vitro ChIP-seq data, we mapped 519 heterozygous SNPs in the promoters of 240 genes to search for the potentially disrupted TF binding sites using motifbreakR. As a result, the binding sites of 787 TFs were predicted to be affected by the identified variants. To identify candidate TFs potentially contributing to inter-individual variation in the response to EMPA, we performed pairwise differential expression analysis using the in vitro RNA-seq data between pairs of individuals displaying the most pronounced differences in EMPA response, as defined by ddASE of the corresponding target genes. A total of 1 384 TF–patient contrast tests were performed, and Benjamini–Hochberg false discovery rate correction was applied across all tests. After correction (adjusted *p* < 0.1; |log2FC| > 0.5), 186 TF–patient comparisons remained significant, corresponding to 146 unique TFs (Supplementary Table S2).

Consistent with our hypothesis, our list of 146 candidate TFs associated with the response to EMPA comprises factors playing important roles in the regulation of immune and inflammatory processes, including those associated with diabetes. First and foremost, these include JUN, JUNB, FOS, FOSB, ATF3, and ATF4, which are components of the AP-1 transcription factor complex, well known as one of the key components of inflammatory signaling pathways [32,33]. Another prominent group is the interferon regulatory factors (IRFs), namely, IRF4, IRF7, and IRF8, as well as the ETV5 TF, which are pivotal in the regulation of immune and inflammatory responses and closely associated with interferon signaling. Notably, AP-1 has been shown to cooperate with IRF4 and IRF8 through binding to AP-1–IRF composite elements (AICEs) [34,35]. Another group comprises several Kruppel-like TFs (KLF2, KLF4, and KLF6), which are involved in immune and inflammatory processes [36]. KLF2 regulates the endothelial nitric oxide synthase (eNOS)/nitric oxide (NO) pathway [36]. KLF4 triggers the VEGF signaling pathway and activates NF-κB signaling [37]. EGR1 (MEK-ERK1/2 signaling pathway) [38], CEBPD (phosphatidylinositol 3-kinase, p38, JAK, JNK, and PKA signaling pathways) [39], HES1 (Notch pathway) [40], NFAT5 (IKK/NF-κB signaling pathway) [41], SOX4 (NF-κB signaling pathway) [42], and TBX21 (IFN-γ-STAT1 and IL-12-STAT4 signaling pathways) [43] were have also been reported to participate in inflammatory responses.

A much larger number of TFs (ATF3, ATF4, ATOH8, CEBPD, EGR1, E2F1, FOS, HES1, HMGA1, IRF4, IRF8, JUN, JUNB, KLF4, KLF5, KLF6, KLF9, KLF11, LEF1, NFAT5, RUNX3, SMAD3, SNAI1, SOX4, TEAD3, TWIST1, ZEB1) from the analyzed list were found to be involved in the processes underlying fibrosis (some of these TFs are concurrently associated with inflammatory signaling), which is one of the key mechanisms in the development of severe diabetic complications and comorbidities: cardiomyopathy, hepatic insufficiency, retinopathy, or peripheral neuropathy [44]. Published evidence for altered expression of many of these TFs in different forms of diabetic fibrosis is summarized in Table 1.

In particular, ATF3, FOS, JUNB, SMAD3, and KLF11 were shown to be associated with diabetic cardiac fibrosis. Notably, whereas increased expression of FOS and JUNB exerts both profibrotic and proinflammatory effects [52], increased ATF3 expression appears to play a protective role [72]. The upregulation of SMAD3 promotes diabetic cardiomyopathy [73], while KLF11 reduces cardiac fibrosis [58].

The association with renal fibrosis was demonstrated for the ATOH8, CEBPD, E2F1, IRF8, KLF4, KLF5, KLF6, and ZEB1 TFs. Previous studies have shown that the upregulation of CEBPD, E2F1, IRF8, KLF5, KLF6, and ZEB1 promotes renal fibrosis in diabetic nephropathy [49,50,56,60,61,71], whereas the overexpression of ATOH8 or KLF4 attenuates renal fibrosis [48,74].

In addition, the available data suggest that increased expression of ATF4 and EGR1 is associated with both cardiomyopathy [21,46] and diabetic nephropathy [51,75], whereas elevated KLF9 expression has been reported in diabetic cardiomyopathy, diabetic nephropathy, and diabetic retinopathy and KLF9 knockdown has been shown to exert protective effects in these conditions [62,63,64].

Of special interest is the fact that the majority of TFs involved in the processes underlying fibrosis (SMAD3, ATF3, ATF4, ATOH8, EGR1, E2F1, FOS, IRF8, JUN, JUNB, KLF5, KLF6, KLF9, KLF11, LEF1, RUNX3, SNAI1, SOX4, TWIST1, and ZEB1) are related to the canonical (SMAD-dependent) transforming growth factor β (TGF-β) signaling pathway. SMAD3 is one of the central transcriptional regulators in this pathway; moreover, its expression level can itself influence the cellular responses induced by TGF-β [73,76]. The remaining TFs of this set are either controlled by the TGF-β signaling pathway or, conversely, regulate this pathway. For several of these TFs, both types of interactions have been reported (Table 2).

Notably, our list of 146 TFs also includes a large group of 52 zinc finger (ZNF) transcription factors, which are classified according to the presence of C_2_H_2_ zinc finger domains in genome annotations. The role of the overwhelming majority of these factors in the regulation of particular processes is currently vague; accordingly, their potential involvement in diabetes and its complications remains to be elucidated.

### 2.3. Application to the In Vivo Dataset Showed Consistency with the In Vitro Data

The findings were further validated in an independent in vivo cohort. Using ChIP-seq profiling for H3K4me3 and H3K27ac histone modifications, we genotyped ten T2DM patients receiving EMPA monotherapy for 353 promoter SNPs associated with allele-specific regulatory effects involving 146 TFs that were identified as differentially expressed in the in vitro EMPA experiment. For each promoter SNP, pairwise comparisons were performed between patients sharing the same genotype at that promoter SNP. Logistic regression was then applied to the in vivo RNA-seq data to evaluate differential allele-specific expression for each gene–patient pair. A total of 1150 comparisons involving 156 distinct genes were analyzed. After Benjamini–Hochberg false discovery rate correction, 202 comparisons remained significant (adjusted *p* < 0.1), corresponding to 85 unique genes exhibiting with inter-individual variation in differential allele-specific expression (ddASE) in response to EMPA (Supplementary Table S3).

We next analyzed the expression of 111 TFs predicted to bind sites disrupted by heterozygous promoter SNPs in these 85 genes. A total of 4 548 TF–patient contrast tests were performed, and Benjamini–Hochberg false discovery rate correction was applied across all tests. After correction (adjusted *p* < 0.1; |log2FC| > 0.5), 1 155 comparisons remained significant, corresponding to 98 unique TFs showing significant expression differences in at least one patient contrast (Supplementary Table S4). Similar to the in vitro results (Supplementary Table S2), this set comprised a large number of TFs implicated in diabetic fibrosis and inflammation.

### 2.4. Determining the Relationship Between the Empagliflozin Effects on TF Expression and Putative Target Genes Allele-Specific Expression in T2DM Patients

To gain deeper insight into the regulatory mechanisms underlying variation in the EMPA response among T2DM patients, we first focused on genes exhibiting treatment-induced differential allele-specific expression (dASE). We then tested whether these changes could be partially explained by concomitant changes in the expression of TFs predicted to regulate the corresponding target genes.

For 261 previously prioritized putatively functional promoter SNPs identified in the in vivo cohort (Figure 1), we modeled the relationship between predicted target gene dASE and changes in TF expression. Of these, 180 SNPs were observed in at least three heterozygous individuals, corresponding to 124 genes and 570 TFs predicted to have binding sites disrupted by these variants based on motif disruption analysis. For each gene–SNP–TF combination, we assessed the linear association between the change in TF expression between day 0 and day 1 and the dASE of the corresponding target gene, defined as the log2 change in allelic imbalance between day 0 and day 1. This analysis identified 16 nominal target gene–TF associations (adjusted *p* < 0.1 after Benjamini–Hochberg correction performed separately for each SNP; Table 3, Supplementary Figure S1) representing candidate EMPA-responsive regulatory interactions that may contribute to inter-individual variation in transcriptional responses to treatment.

According to the published literature, the majority of the predicted target genes are involved in key mechanisms underlying T2DM pathogenesis and its complications, including chronic inflammation (*SLC20A1* [85], *BMP8B* [86], and *PFKFB3* [87]), insulin resistance (*DNAJB1* [88] and *SLC20A1* [85]), metabolic reprogramming (*SULF2* [89] and *PFKFB3* [87]), fibrotic remodeling (*SULF2* [90], *EMP3* [91], *BMP8B* [86], and *LILRB4* [92]), diabetic kidney disease (*DFFB* [93], *SULF2* [94], *SLC20A1* [95], *ADAM10* [96], and *PFKFB3* [87]), renal fibrosis (*EMP3* [91]), diabetic cardiomyopathy (*AMPD3* [97] and *PFKFB3* [87]), diabetic neuropathy (*MBP* [98,99]), and non-alcoholic steatohepatitis (*BMP8B* [86]).

The *PFKFB3* gene, which encodes a master regulatory enzyme of glucose metabolism, is of the greatest interest. Recent studies have demonstrated that PFKFB3 plays an important role in the development of diabetic cardiomyopathy, diabetic kidney disease, and diabetic retinopathy through its involvement in inflammatory signaling, fibrosis, endothelial dysfunction, and macrophage metabolic reprogramming [87]. Importantly, SGLT2 inhibitors have been shown to modulate macrophage polarization and inflammatory activation by suppressing glycolysis through the downregulation of PFKFB3 [26].

Another putative target gene, *EMP3* (epithelial membrane protein 3), is a key player in the development of renal fibrosis, facilitating the activation of the TGF-β/SMAD3 signaling pathway. It promotes fibrosis by directly interacting with TGF-β receptor 2 (TGFBR2), which subsequently activates the TGF-β/SMAD3 pathway [91]. In contrast, the corresponding TF, TCF4, represses TGF-β signaling and inhibits endothelial-to-mesenchymal transition [100].

DNAJB1, a member of the HSP40 chaperone family, has been associated with insulin resistance and decreases with glucose normalization in T2D [88]. Moreover, in patients with heart failure, DNAJB1 protein levels were significantly altered by EMPA treatment [101].

LILRB4 is an inhibitory immune receptor that alleviates cardiac fibrosis and inflammation through the regulation of NF-κB and MAPK signaling [102], and its knockdown promotes fibrosis, inflammation and apoptosis [92]. In our analysis, *LILRB4* dASE was associated with changes in the expression of SMAD3, a key mediator of profibrotic TGF-β/SMAD3 signaling [83].

ADAM10, a Notch pathway protease, is upregulated in diabetic nephropathy [96]. In podocytes, *ADAM10* knockdown protects against high-glucose-induced injury by inhibiting MAPK-mediated pyroptosis [103]. The associated TF RARA is downregulated by TGF-β1, suggesting crosstalk between TGF-β1/SMAD and retinoic acid signaling [104].

Upregulated AMPD3 contributes to contractile dysfunction in diabetic cardiomyopathy [97]. The associated TF NFKB2 is a component of the NF-κB signaling pathway, which plays a pivotal role in renal fibrosis [105].

The remaining TFs found in our analysis, many of which are poorly characterized, may represent as yet-unrecognized mediators of the transcriptional response to EMPA. It should be noted, however, that multiple-testing correction was performed separately for each promoter SNP. When a global multiple-testing correction was applied across all tested gene–SNP–TF associations, none of the reported associations remained significant. Therefore, these findings should be considered nominal associations that require validation in independent cohorts.

## 3. Discussion

Despite the decades of intensive research efforts, the molecular genetic basis of the events involved in T2DM pathogenesis is still poorly understood and the treatment is still challenging [106]. There is a significant burden of advanced disease complications, such as pathologic cardiac remodeling, renal failure, hepatic insufficiency, retinopathy, or peripheral neuropathy, which determine the disease severity and mortality. All these conditions have been epidemiologically associated with accelerated fibrosis [44,107], yet the insights are often drawn from the concepts developed in the context of other fibrotic disorders. Therefore, the study of T2DM molecular pathology is of great significance for the clinical treatment of the disease.

Recent advances have led to new strategies based on omics data, mainly transcriptomic, for comprehensive understanding of key pathological processes and the identification of potential therapeutic targets. The analysis of allele-specific expression (ASE) in RNA-seq data is a useful and sensitive approach for investigating the genetic or epigenetic factors that may cause the variation in traits. ASE refers to the imbalance in the transcription between two alleles at a heterozygous locus within the same individual and hence performed in a consistent genetic background and under equivalent environmental conditions. As a result, it overcomes a major challenge in human genetics: between-subject variability. ASE analysis has been widely applied to human subjects aiming to clarify the pathogenesis of complex diseases [108], as well as to important farm animals and crops to link the expression biases to specific traits [109,110]. The results of ASE analysis are often supplemented with the results of other techniques, for example, genome-wide association study (GWAS), in order to examine the allelic imbalances potentially determined by trait-associated variants [111]. An emerging area is the study of differential allele-specific expression (dASE) between individuals, tissues, or conditions to gain the novel insight into the context-specific gene regulation [112], including the response to drugs and the effects of various pathogens. The most illustrative example here is the study by Moyerbrailean et al. [113] on the allele-specific effects in transcriptomic data derived from five cell types of three individuals when exposed to 50 different treatments comprising nutrients, common drugs, steroid and peptide hormones, metal ions, and environmental contaminants.

In this study, we present a sophisticated computational framework aimed to evaluate the transcription factors most likely contributing to the mechanisms underlying the effects of the antidiabetic drug empagliflozin (EMPA). Within this workflow, two human PBMC RNA-seq datasets generated in vitro (*n* = 3) and in vivo (*n* = 10), were integrated. We used dASE analysis as the leading component of a prioritization strategy to narrow the search for regulatory TFs.

To obtain the first list of putative EMPA-treatment-associated TFs, we performed in vitro experiments using PBMCs from three healthy donors exposed to 10 nM EMPA for 24 h (see Section 4). RNA-seq data (collected at baseline and after 1 day of EMPA treatment) and baseline ChIP-seq data (before EMPA treatment) were generated and analyzed (see Figure 1 for pipeline details). This approach allowed us to reveal 146 TFs potentially involved in the cellular response to EMPA and not associated with its systemic effects (first and foremost, glycemic).

As has emerged, the largest group of TFs from the list was associated with the regulation of the processes underlying diabetic fibrosis (Table 1), which plays a key role in the development of severe T2DM complications, including cardiomyopathy, nephropathy, hepatic insufficiency, and retinopathy [44]. Notably, among the TFs implicated in fibrotic processes, a distinct group of factors (SMAD3, ATF3, ATF4, ATOH8, EGR1, E2F1, FOS, IRF8, JUN, JUNB, KLF5, KLF6, KLF9, KLF11, LEF1, RUNX3, SNAI1, SOX4, TWIST1, and ZEB1) emerged that are associated, directly or indirectly, with the canonical (SMAD-dependent) TGF-β signaling pathway (Table 2).

In particular, increased expression of genes encoding ATF3, ATF4, E2F1, FOS, JUN, JUNB, IRF8, KLF6, KLF11, LEF1, SNAI1, TWIST1, and ZEB1 with the activation of the TGF-β/SMAD3 pathway has been reported in the relevant literature; moreover, many of these TFs have been shown to induce the expression of profibrogenic factors, such as α-SMA (α-smooth muscle actin), COL1A1 and COL3A1 (collagen types I and III), and FN1 (fibronectin) [56,58,61,76,77,78,79,83,114].

The ability to act as upstream regulators in the TGF-β signaling has been shown for ATF3, KLF6, and EGR1. In particular, ATF3 enhances TGFBR2 transcription by binding to a cognate site in the TGFBR2 promoter. After TGF-β stimulation, the TGF-β type II receptor forms a tetraheteromeric complex with the TGF-β type I receptor, leading to TGFBR1 activation via phosphorylation. Then, the activated TGFBR1 phosphorylates SMAD2 or SMAD3, thereby activating the TGF-β/SMAD pathway, and increases α-SMA, collagen I, and vimentin production [76,115]. KLF6 is able to bind to the SMAD3 promoter and activate *SMAD3* transcription [61]. EGR1 also upregulates profibrotic genes by inducing *PAR1* expression, encoding a G-protein coupled receptor that stimulates the TGF-β/SMAD pathway [51].

A number of TFs are known to regulate the target genes of the TGF-β/SMAD3 signaling pathway via protein–protein interactions with SMAD3. These TFs are known as SMAD cofactors and more than 40 such factors have been identified to date using bioinformatic, biochemical, and molecular biological approaches [76]. Among the TFs identified in our study, ATF3, ATOH8, JUN, JUNB, KLF5, LEF1, RUNX3, SNAI1, SOX4, and ZEB1 have been associated with the canonical TGF-β1/SMAD pathway. Most of them enhance SMAD transcriptional regulatory activity, whereas ATOH8 binds to SMAD3 to form a repressive transcriptional complex downregulating the target genes of the TGFβ–SMAD2/3 signaling [76,115]. KLF11 has been reported to exhibit similar properties to ATOH8 [81].

Thus, according to published data, numerous TFs from the list are associated with the TGF-β/SMAD canonical profibrotic signaling cascade. This suggests a high potential of the SGLT2is impact on the expression/activation of these TFs in implementing the protective effects in diabetic fibrosis [116,117]. The experimental data for some of these TFs favor this assumption. In particular, EMPA was shown to considerably improve the cardiac function, in particular, decreasing the mRNA levels of *ATF4*, in a model of diabetic cardiomyopathy in the streptozotocin-induced diabetic rats [118]. Using a model of isoprenaline-induced cardiac fibrosis in nondiabetic rats, a considerable decrease in SMAD3 expression as a component of EMPA cardioprotective effect was demonstrated [119]. Our results considerably expand the list of potential targets for antifibrotic effects of EMPA and other SGLT2is. Our data are also helpful when designing and assessing novel antidiabetic drugs.

Overall, the in vivo data on TFs predicted to participate in the regulation of gene expression in response to EMPA were largely consistent with the corresponding in vitro findings (Supplementary Tables S2 and S4). In addition, the in vivo dataset was used to identify novel target genes of TFs potentially implicated in the EMPA response. Using promoter SNPs prioritized in the in vivo cohort, we modeled the associations between changes in TF expression and corresponding treatment-induced changes in allele-specific expression (dASE) of their predicted target genes. This analysis identified 16 nominal TF–target gene associations, suggesting that the magnitude of the allele-specific transcriptional response to EMPA is influenced by the expression levels of TFs regulating these genes. These associations were supported by per-SNP multiple-testing correction (Benjamini–Hochberg adjusted *p* < 0.1) but did not retain statistical significance after global correction across all tested gene–SNP–TF associations. Therefore, these findings should be interpreted as candidate regulatory relationships requiring further investigation. The identified potential target genes included genes associated with processes and signaling pathways involved in the progression of metabolic disorders and T2DM complications, including inflammatory signaling, oxidative stress responses, and fibrotic remodeling. Notably, several TFs associated with TGF-β-mediated pathways, including KLF9, TCF4, SMAD3, and RARA, were among the candidate regulators.

Despite these promising findings, several limitations should be acknowledged. First, the relatively small sample cohort size is a technical limitation. Thus, in vivo RNA-seq samples obtained from the same patient at four time points were treated as biological replicates in the DESeq2 analysis although being not fully independent samples. That may compromise the robustness of differential expression calls, potentially introducing bias when prioritizing candidate TFs. Expanding the analysis to a larger number of subjects is desirable, as it enables the derivation of broadly applicable findings. This is also important given that allele-specific event analysis is inherently restricted to heterozygous sites. However, the criteria for sample selection are essential, as any time-dependent fluctuations in clinical status, medication, and achieved glycemic control could introduce variability into the observed transcriptional dynamics. Another shortcoming to note is the heterogeneous composition of the analyzed cells. PBMCs comprise monocytes and lymphocytes, and the use of bulk PBMCs may mask cell-subtype-specific signals. However, cell isolation strategies themselves unquestionably influence cellular transcriptional activity [120]. Moreover, PBMCs may not fully represent the molecular landscape of key metabolic tissues (i.e., adipose tissue, pancreatic islets, and liver). Nonetheless, despite these limitations, PBMCs provide a widely used practical model for investigating systemic inflammation and immune-metabolic disturbances (which are considered key drivers of T2DM progression), including in omics studies [121,122,123]. Additionally, PBMCs are can be obtained through a minimally invasive procedure.

Given the aforementioned limitations, this study, however, provides a detailed exploratory analysis aimed to identify TFs whose expression changes correlate with treatment response by integrating allele-specific expression with transcription factor binding predictions. The findings are consistent with prior research demonstrating the anti-inflammatory and antifibrotic effects of empagliflozin (EMPA) in preventing and treating severe T2DM complications [9,10,11,12]. The identified lists of transcription factors involved in these processes can therefore serve as a valuable resource for analyzing other omics datasets related to the antidiabetic effects of SGLT2 inhibitors and for identifying promising targets for novel antidiabetic therapies. Overall, our results show that integrating multi-omics data can capture biologically meaningful allele-specific changes concerning the changes in TF expression and TF activity in immune cells underlying therapy response. Because our approach is relatively affordable and scalable, it provides a foundation for further research, including mechanistic cell- and tissue-specific studies, single-cell omics analyses, and functional validation of the key predicted TFs and TF–target gene interactions.

## 4. Materials and Methods

### 4.1. Study Design and PBMC Isolation and Sampling

The in vitro effects of EMPA on human PBMCs were assessed in a cohort of three healthy volunteers (one man and two women; median age, 48; see Supplementary Table S5 for basic anonymized data). Blood (50 mL) was sampled from each donor to isolate PBMCs by density gradient centrifugation using Ficoll Diacoll-1077 (Dia-M, Novosibirsk, Russia) as previously described [124]. The freshly isolated PBMCs were suspended in RPMI-1640 medium and incubated in vitro as described below.

The cohort for in vivo study comprised 10 T2DM patients (five men and five women aged 44–65 years; median age, 60) of the Research Clinic with the Institute of Clinical and Experimental Lymphology (branch of the Institute of Cytology and Genetics, Siberian Branch, Russian Academy of Sciences) who received a 90-day EMPA treatment (10 mg/day). Inclusion and exclusion criteria, as well as the clinical data, are available in Korbut et al. [125]. Notably, a significant hypoglycemic effect of treatment was demonstrated in the cohort and the data also suggested a protective effect of EMPA against low-grade inflammation. Basic anonymized characteristics and a number of clinical parameters at the baseline are presented in the Supplementary Tables S5 and S6, respectively. The baseline blood samples of 50 mL and follow-up blood samples of 30 mL on days 1, 30 and 90 of EMPA therapy were collected and assayed. PBMCs were isolated by Ficoll density gradient centrifugation; the PBMC samples from days 1, 30 and 90 of the therapy were used for RNA extraction; the PBMC samples from day 0 were divided into two aliquots for RNA extraction and ChIP workflow.

### 4.2. In Vitro Study: PBMC Cultivation Conditions

PBMCs at a concentration of 1 × 10^6^/mL were seeded in the T75 flasks with RPMI-1640 (L-glutamine and sodium bicarbonate; BioLot, Saint Petersburg, Russia) supplemented with 10% FCS (Capricorn Scientific, Ebsdorfergrund, Germany) and 1% penicillin/streptomycin (BioLot, Russia). The cells were incubated at 37 °C and 5% CO_2_ in the culture media containing EMPA (Selleck Chemicals, Houston, TX, USA) at a final concentration of 10 nM in 0.1% DMSO or 0.1% DMSO as a control for 24 h. Subsequently, the control cells for each donor were divided into two aliquots: one for RNA extraction and the other for the ChIP-seq procedure; the EMPA-treated cells were used for RNA extraction.

### 4.3. RNA Extraction and RNA-Seq Library Preparation

Total RNA was extracted using FreeZol (Vazyme, Nanjing, China); polyA fraction was purified using NEBNext Poly(A) mRNA Magnetic Isolation Module (New England Biolabs, Ipswich, MA, USA) according to the manufacturer’s instructions. The libraries were generated using the NEBNext^®^ Ultra^™^ II Directional RNA Library Prep Kit for Illumina^®^ (New England Biolabs, Ipswich, MA, USA). Six RNA-seq libraries were prepared as part of an in vitro experiment for three donors and two conditions (EMPA treatment and control) and 40 RNA-seq libraries, for ten individuals and four conditions (0, 1, 30, and 90 days), for an in vivo experiment. The RNA-seq data obtained for days 0 and 1 were used to search for allele-specific expression-associated changes; the RNA-seq data for days 30 and 90 were included in pairwise differential expression analyses (see Section 4.9 and Section 4.11 for details). The DNA concentration of each library was determined with a Qubit fluorometer (Thermo Fisher Scientific, Waltham, MA, USA) and DNA size distribution, with a QSEP100 system (BiOptic Inc., New Taipei City, Taiwan).

### 4.4. Chromatin Immunoprecipitation (ChIP) and ChIP-Seq Library Preparation

To discriminate the active promoter regions, ChIP sequencing was performed for profiling two activating histone modifications, H3K4me3 and H3K27ac, in both in vitro and in vivo experimental steps [126].

To preserve the protein–DNA interactions, PBMCs were fixed with 1% formaldehyde (Sigma, Saint Louis, MO, USA) in phosphate-buffered saline (PBS) at a room temperature for 9 min. After fixation, the cells were quenched with 125 mM glycine for 5 min, washed twice with 1× PBS, and resolved in 800 µL of ice-cold lysis buffer, containing 10 mM Tris pH 7.5, 1 mM EDTA, 0.5% SDS, 0.1% sodium deoxycholate, 1% Triton X-100, and protease inhibitor cocktail (Thermo Fisher Scientific, Waltham, MA, USA). The cells were immediately sheared to generate the chromatin fragments of 150–600 bp using a BANDELIN homogenizer (BANDELIN electronic GmbH & Co. KG, Berlin, Germany) with additional cooling on ice. See Damarov et al. 2024 [124] for the details of the optimized ChIP protocol. Briefly, the chromatin fragments prepared from approximately 10^8^ cells were incubated in a total volume of 300 µL containing ChIP buffer (10 mM Tris pH 7.5, 1 mM EDTA, 0.1% SDS, 0.1% sodium deoxycholate, 1% Triton X-100, and 100× protease inhibitor cocktail) and the protein G–coated magnetic beads pretreated with 8 µg of the antibodies to H3K27ac or H3K4me3 (Abcam, Cambridge, UK) at 4 °C overnight with gentle rotating. Then, the probe was sequentially washed twice with 1 mL of each solution: lysis buffer, high salt buffer (10 mM Tris pH 7.5, 1 mM EDTA, 0.1% SDS, 0.1% sodium deoxycholate, 1% Triton X-100, and 0.5 M NaCl), TE buffer (10 mM Tris pH 7.5, 1 mM EDTA, and 1% Triton X-100), and the TE buffer without Triton X-100. The remaining steps of ChIP, including elution, cross-linking reversal, and DNA purification were performed as earlier described [124].

The ChIP-seq libraries were prepared from 1–5 ng of ChIP DNA using the NEBNext Ultra II DNA Library Prep Kit for Illumina (New England BioLabs, Ipswich, MA, USA). End prep, adaptor ligation, cleanup of adaptor-ligated DNA, PCR enrichment of adaptor-ligated DNA (8–10 cycles), and cleanup of PCR reaction steps were performed according to the manufacturer’s protocols. In total, 26 ChIP-seq libraries were constructed: six libraries for two histone marks and three individuals for the in vitro experiment and twenty libraries for two histone marks and PBMCs of ten T2DM patients sampled on day 0 of the in vivo experiment. DNA concentration of each library was determined with a Qubit Fluorometer (Thermo Fisher Scientific, USA) and DNA size distribution, by running a sample on a QSEP100 system (BiOptic Inc, Taiwan).

### 4.5. Illumina Sequencing

Multiplexing was performed using NEBNext^®^ Multiplex Oligos for Illumina, Index Primers Set 1 (New England Biolabs, USA) to yield approximately 116.0 ± 23.5 million reads for each RNA-seq library and 75.2 ± 9.7 million reads for each ChIP-seq library. Cluster generation and paired-end 150 bp sequencing were conducted with standard Illumina protocols by Evrogen (Moscow, Russia) on the Novaseq 6000 (Illumina, San Diego, CA, USA).

### 4.6. Data Preprocessing and Quality Control

Raw data were processed with fastp (v0.20.1) to remove adapter sequences and reads shorter than 36 nt [127]. The filtered reads were aligned to the human reference genome (GRCh38/hg38) using HISAT2 (v2.2.1) [128] with a genome index incorporating the known SNPs and annotated transcripts to minimize reference allele mapping bias. Alignments with MAPQ < 30 were removed using samtools (v1.21) [129].

### 4.7. Identification and Annotation of Heterozygous SNPs

For each donor, heterozygous SNPs were identified using the sequencing reads pooled across all ChIP-seq and RNA-seq samples available for the individual. Variant calling was performed with bcftools mpileup (v1.21), retaining SNPs with a minor allele fraction > 0.1 and read depth > 20. The variants located on sex chromosomes and alternative contigs were excluded. The SNPs were annotated using the SNPlocs.Hsapiens.dbSNP155.GRCh38 database [130].

### 4.8. Determination of the Gene List for Analysis

Based on the in vitro ChIP-seq data, the genes were included in downstream analyses if they met the following criteria: (i) at least one heterozygous SNP was present within the promoter region (−2000 to +200 bp relative to the transcription start site) and (ii) the same heterozygous SNP was shared by at least two individuals from the in vitro group.

The patients in the in vivo cohort were genotyped for the promoter SNPs identified in the in vitro analysis. Thus, the in vivo validation analyses were restricted to genes containing at least one of the in vitro-derived heterozygous promoter SNPs and showing evidence of inter-individual differences in treatment-induced changes in allelic imbalance in the in vitro RNA-seq data.

### 4.9. Search for Allele-Specific Expression-Associated Changes

For allele-specific expression (ASE) analyses, heterozygous exonic SNPs within the corresponding genes with a minimum coverage of 50 RNA-seq reads were selected. Promoter and exon coordinates were obtained from the TxDb.Hsapiens.UCSC.hg38.knownGene annotation [131]. The genes located within the *HLA* locus were excluded due to high mapping error rates and unreliable allele frequency estimates [132].

For each SNP, the allele with higher coverage was defined as the major allele and the other as the minor allele. When multiple informative SNPs were present within a gene, the allele-specific counts were aggregated across SNPs by averaging the major- and minor-allele read counts for each individual.

*Differential allele-specific expression* (dASE), reflecting the change in allelic imbalance between EMPA-treated and baseline conditions, was calculated as

$$dASE={log}_{2}\left(\frac{{ASE}_{1}}{{ASE}_{0}}\right),$$

where ${ASE}_{i}$ denotes the allele-specific expression of a gene in condition *i* and is defined as the ratio of major- to minor-allele read counts, ${ASE}_{i}=\frac{{maj}_{i}}{{min}_{i}}$.

*Inter-individual variability in differential allele-specific expression* (ddASE), reflecting whether the effect of treatment on allelic imbalance differs between individuals, was assessed for each gene using a generalized linear model with a binomial distribution:

is_major ~ condition × person,

where *is_major* indicates allele identity (major = 1, minor = 0), *condition* denotes baseline or EMPA-treated samples, and *person* denotes the two individuals sharing a heterozygous promoter SNP for the gene.

The major- and minor-allele read counts from all samples were incorporated as observation weights. The interaction term (condition × person) captures individual-specific differences in the allelic response to treatment. The genes were classified as exhibiting significant ddASE if at least one pairwise comparison yielded a significant interaction term after Benjamini–Hochberg correction (adjusted *p* < 0.1).

### 4.10. Identification of Potentially Disrupted TF Binding Sites

Putative TF binding sites disrupted by nucleotide substitutions were identified using motifbreakR (v2.22.0) (*p* < 10^−4^) [31]. The TF binding site annotations were extracted from the HOCOMOCOv13 database [133].

### 4.11. Identification of Differentially Expressed Genes

The RNA-seq reads were quantified using featureCounts (v2.1.1) [134] with gene annotations from Ensembl release 115 [135].

Gene expression normalization and differential expression analysis were conducted using DESeq2 (v1.47.5) [136], excluding low-count transcripts. The objective here was to compare transcription factor expression between individuals with contrasting dASE responses, and therefore patient identity was used as the design factor (design = ~ patient). RNA-seq samples collected at multiple time points were treated as biological replicates representing each donor’s expression profile across the study period.

For the in vitro cohort, samples collected at two time points (days 0 and 1) were included, whereas for the in vivo cohort, samples collected at four time points (days 0, 1, 30, and 90) were included. For each tested pairwise patient contrast, gene-level differential expression statistics were extracted from the fitted model, and the resulting *p* values were corrected using a global Benjamini–Hochberg procedure across all tested patient–gene contrasts. Genes with adjusted *p* < 0.1 and |log2FC| > 0.5 were regarded as differentially expressed.

### 4.12. Association Between Transcription Factor Expression Changes and dASE of Target Genes

TF expression was quantified in baseline (day 0) and treatment (day 1) samples using DESeq2-normalized counts. Log2-transformed expression values were used to calculate TF expression change (ΔTF) per patient as the difference in log2-normalized expression between day 1 and day 0.

For each SNP, associations between TF expression changes and dASE of the corresponding target genes were evaluated using the linear model:

dASE ~ β_0_ + β_1_ × ΔTF

Multiple-testing correction was performed independently for each promoter SNP across its corresponding TF–gene pairs, as well as globally across all SNP-TF-gene combinations, using the Benjamini–Hochberg procedure. Associations with adjusted *p* value < 0.1 were considered significant. To assess whether the observed association was driven by any individual donor, we also performed a leave-one-out sensitivity analysis in which the regression model was refitted after sequentially excluding each donor.

### 4.13. Data Analysis Environment

Analyses were performed in the R programming environment (v4.5.1) if not specified otherwise.

### 4.14. Gene and Protein Nomenclature

Gene and protein symbols were standardized throughout the manuscript and Supplementary Materials according to current human nomenclature conventions using HGNC-approved gene symbols.

## Figures and Tables

**Table 1:** Candidate transcription factors identified by in vitro analysis of empagliflozin response with reported roles in diabetic fibrosis.

TF	Function	Role in Diabetic Fibrosis	Experimental Model	Source
ATF3	Regulator of cellular stress response	Protective role in DCM	Mutant mice with a cardiac-specific ATF3 deficiency, fed a high-fat diet for 15 weeks (cardiomyocytes)	[45]
ATF4	Regulator of cellular stress response and amino acid metabolism	Promotes the progression of diabetic cardiac fibrosis	STZ-treated mice and mice with induced myocardial infarction (heart tissues); high-glucose-exposed HL-1 cells (mouse cardiomyocytes)	[46]
		Promotes renal tubulointerstitial fibrosis by suppressing autophagy in DN	Heterozygous *ATF4* knockout mice with STZ-induced DN (renal tissues); NRK-52E cells, cultured in high glucose (kidney epithelial-like cells)	[47]
ATOH8	Regulator of cellular differentiation, organ development, stress responses, and iron metabolism	Overexpression restrains renal fibrosis in DN	High-glucose-treated mouse mesangial cells and db/db mouse kidneys	[48]
CEBPD	Regulator of immune and inflammatory responses, cell proliferation, and apoptosis	Positively associated with DN progression through transcriptional activation of HIF1A, a driver of renal fibrosis	Human kidney tubulointerstitial biopsy transcriptomes from DN patients	[49]
E2F1	Regulator of cell cycle progression and proliferation	Promotes renal tubular fibrosis and cellular senescence in DKD	db/db mice and high-glucose-treated mouse renal tubular epithelial cells	[50]
EGR1	Immediate-early regulator of growth factor responses and tissue remodeling	Contributes to the development of renal tubulointerstitial fibrosis in DKD	OLETF rat model of T2DM, high-glucose-treated HK-2 human proximal tubular cells	[51]
FOS	Component of AP-1 transcription factor complex	Involved in MAPK-associated regulation of DCM	Non-obese diabetic rats, high-glucose-treated H9c2 cardiomyocytes	[52]
HES1	Transcriptional effector of the Notch signaling pathway	Upregulated in DN	Human podocytes, growth hormone-treated mice, and kidney biopsies from patients with DN	[53]
HMGA1	Architectural transcription factor regulating chromatin organization	Promotes cardiac remodeling and dysfunction in DCM	STZ-induced diabetic mice and high-glucose-treated neonatal rat cardiomyocytes	[54]
IRF4	Regulator of immune system development and function	Promotes renal fibrosis and extracellular matrix remodeling in DN	High-glucose-treated human podocytes (AB8/13), STZ-induced diabetic rats, human DN transcriptomic datasets	[55]
IRF8	Regulator of immune system development and function	Upregulation promotes renal fibrosis in DN	NRK-49F renal fibroblasts, UUO-induced mouse renal fibrosis model, human chronic kidney disease datasets	[56]
JUN	Component of AP-1 transcription factor complex	Activates TGF-β1 transcription and contributes to renal fibrosis in DN	Human renal mesangial cells and db/db mice with DN	[57]
JUNB	Component of AP-1 transcription factor complex	Involved in MAPK-associated regulation of DCM	Non-obese diabetic rats, high-glucose-treated H9c2 cardiomyocytes	[52]
KLF11	Regulator of cell proliferation, apoptosis, inflammation, and metabolism	Attenuates cardiac fibrosis by suppressing profibrotic gene expression	Cardiac-specific *KLF11* transgenic mice subjected to transverse aortic constriction; mouse and human failing hearts	[58]
KLF4	Regulator of cellular differentiation and anti-inflammatory responses	Attenuates TGF-β1-induced inflammation in DN and is downregulated in diabetic kidneys	Human proximal tubular HK-2 cells exposed to high glucose or TGF-β1; STZ-induced diabetic mice	[59]
KLF5	Regulator of cell proliferation and tissue remodeling	Promotes EMT and renal fibrosis in DKD by repressing CDH1 expression	Renal tubular epithelial cells, db/db mice, kidney-specific *KLF5* knockdown mice	[60]
KLF6	Regulator of tissue repair, cell growth, differentiation, and inflammation	Upregulation promotes renal fibrosis in DN	TGF-β1-stimulated mouse renal tubular TCMK1 cells and mice with UUO	[61]
KLF9	Regulator of cellular stress and differentiation responses	Promotes cardiac fibrosis and inflammation in DCM	STZ-induced diabetic mice and high-glucose-treated neonatal rat cardiomyocytes	[62]
		Promotes high glucose-induced podocyte injury, inflammation, and oxidative stress in DN	High-glucose-treated human podocytes; serum samples from patients with DN	[63]
		Promotes pathological retinal angiogenesis in diabetic retinopathy	High-glucose-treated human retinal microvascular endothelial cells and oxygen-induced retinopathy rats	[64]
LEF1	Transcriptional mediator of Wnt/β-catenin signaling	Identified as a regulator of a majority of differentially expressed genes in DN	Human diabetic and healthy glomerular microarray datasets	[65]
NFAT5	Regulator of osmotic stress and immune system responses	Promotes renal fibrosis in DN; *NFAT5* knockdown attenuates extracellular matrix accumulation	High-glucose-treated mouse mesangial cells and db/db DN mice	[66]
RUNX3	Regulator of cell cycle progression, immune cell development, and tumor suppression	Reduces renal fibrosis in DN through activation of the Apelin/SIRT1/FOXO pathway	STZ-induced DN mice; high-glucose-treated mesangial cells	[67]
SMAD3	Driver of fibrotic and extracellular matrix gene expression	Principal mediator of the profibrotic effects of TGF-β signaling in renal fibrosis	TGF-β1-stimulated mouse renal tubular TCMK1 cells and mice with UUO	[61]
SNAI1	Transcriptional repressor regulating EMT	Promotes EMT and extracellular matrix accumulation in diabetic renal tubulointerstitial fibrosis	NRK-52E renal tubular epithelial cells, db/db diabetic mouse model	[68]
SOX4	Regulator of development and EMT	Promotes renal fibrosis and mesangial cell proliferation in DN	STZ-induced DN mice; high-glucose-treated human mesangial cells	[69]
TWIST1	Regulator of embryonic development, tissue differentiation, cell migration, and EMT	Promotes DN progression by driving EMT and renal tubulointerstitial fibrosis	HK2 renal epithelial cells, HEK293 cells; STZ-induced type 1 diabetic rat model; urine and kidney samples from DN patients	[70]
ZEB1	Regulator of EMT, development, and immune responses	Upregulated in vitamin D-deficient DKD and linked to increased renal EMT and fibrosis	Rat model of STZ-induced type 1 DKD with vitamin D deficiency	[71]

Abbreviations: TF, transcription factor; DCM, diabetic cardiomyopathy; DN, diabetic nephropathy; STZ, streptozotocin; DKD, diabetic kidney disease; UUO, unilateral ureter obstruction; EMT, epithelial-to-mesenchymal transition; EndMT, endothelial-to-mesenchymal transition.

**Table 2:** Candidate transcription factors identified by in vitro analysis of empagliflozin response with reported links to the TGF-β/SMAD signaling pathway.

TF	Function	Link to TGF β/SMAD Pathway	Experimental Model	Source
ATF3	Regulator of cellular stress response	Activates TGF-β signaling and is induced in a TGF-β/SMAD3-dependent manner, forming a positive feedback loop	Human HSCs line LX-2, HEK-293T, human fibrotic liver tissues from patients with hepatic haemangioma, a liver fibrosis mice model	[77]
ATF4	Regulator of cellular stress response and amino acid metabolism	Induced by TGF-β1 through SMAD3 and mTORC1 signaling to promote serine-glycine biosynthesis and collagen production	Primary human lung fibroblasts, human idiopathic pulmonary fibrosis lung tissue	[78]
ATOH8	Regulator of cellular differentiation, organ development, stress responses, and iron metabolism	SMAD3 binds a potential tumor suppressor ATOH8 to form a transcriptional complex that directly represses a series of cell cycle-promoting genes	Human epithelial and lung cancer cell lines, mouse models	[76]
E2F1	Regulator of cell cycle progression and proliferation	Induced by TGF-β and promotes TGF-β1-induced fibroblast-to-myofibroblast differentiation through transcriptional activation of *CCNE2*	Human cardiac fibroblasts	[79]
EGR1	Immediate-early regulator of growth factor responses and tissue remodeling	Activates TGF-β1/SMAD pathway via transcriptional upregulation of PAR1	Rat model of T2DM (OLETF strain), high-glucose-treated HK-2 human proximal tubular cells	[51]
FOS	Component of AP-1 transcription factor complex	Induced by TGF-β and cooperates with SMAD complexes to activate transcription in response to TGF-β	Transiently transfected COS-1 cells	[80]
IRF8	Regulator of immune cell differentiation	Induced by TGF-β1 during renal fibrogenic responses and identified as a potential regulator of renal fibrosis	NRK-49F renal fibroblasts, UUO-induced mouse renal fibrosis model, human chronic kidney disease datasets	[56]
JUN	Component of AP-1 transcription factor complex	Induced by TGF-β and cooperates with SMAD complexes to activate transcription in response to TGF-β	Transiently transfected COS-1 cells	[80]
JUNB	Component of AP-1 transcription factor complex	Induced by TGF-β and cooperates with SMAD2/3 to activate late invasion-associated target genes	Breast and lung cancer cell lines, zebrafish xenograft models	[76]
KLF11	Regulator of cell proliferation, apoptosis, inflammation and metabolism	Induced by TGF-β and cooperates with SMAD3 to repress c-MYC transcription	Human epithelial cell lines (HEK-293, MCF-10A), mouse mammary epithelial cells (EpH4), pancreatic cancer cell lines	[81]
KLF5	Regulator of cell proliferation and tissue remodeling	TGF-β/SMAD cofactor that is acetylated upon TGF-β signaling, switching it into a SMAD-associated transcriptional activator at target promoters	TGFβ-treated epithelial cell lines (HaCaT, HepG2, HEK-293, COS-1)	[76]
KLF6	Regulator of tissue repair, cell growth, differentiation, and inflammation	Induced by TGF-β; an upstream regulator of TGF-β signaling and a positive regulator of *SMAD3* transcription	TGF-β1-stimulated mouse renal tubular TCMK1 cells and mice with UUO	[61]
KLF9	Regulator of cellular stress and differentiation responses	Upregulates *SNX5* expression, which suppresses TGF-β-induced epithelial–mesenchymal transition and invasion	Clear cell renal cell carcinoma cell lines, nude mouse xenograft models	[82]
LEF1	Transcriptional mediator of Wnt/β-catenin signaling	Induced by TGF-β; physically interacts with SMAD3 and cooperates with TGF-β signaling to activate target gene transcription	HepG2, COS-1, HEK293T cells; *Xenopus* promoter reporter assays	[76]
RUNX3	Regulator of cell cycle progression, immune cell development, and tumor suppression	Cooperates with SMADs to mediate TGF-β-dependent growth inhibition	Gastric cancer cell lines (SNU16, MKN28), HEK293 cells, mouse and human gastric epithelium	[76]
SMAD3	Driver of fibrotic and extracellular matrix gene expression	Canonical mediator required for TGF-β signaling	Human epithelial and lung cancer cell lines, mouse models, human lung cancer clinical samples	[76]
SNAI1	Transcriptional repressor regulating epithelial–mesenchymal transition	Induced by TGF-β; forms a transcriptional repressor complex with SMAD3/4 to repress epithelial gene expression during TGF-β-induced EMT	Breast epithelial cell lines (NMuMG, EpH4, EpXT), mouse breast carcinoma model, human breast carcinomas	[76,83]
SOX4	Regulator of development and epithelial–mesenchymal transition	Acts as a SMAD3 cofactor to regulate TGF-β-induced expression of genes involved in migration and extracellular matrix remodeling	Breast cancer cell lines (MCF-7, MDA-MB-231, HCC1954), human mammary epithelial cells, HEK293T cells	[76]
TWIST1	Regulator of epithelial–mesenchymal transition	Induced by TGF-β; promotes TGF-β signaling by stabilizing MEF2A, which directly activates *TBR1* transcription in keloid fibroblasts	Human keloid-derived fibroblasts, normal dermal fibroblasts, keloid tissue samples	[83,84]
ZEB1	Regulator of epithelial–mesenchymal transition, development, and immune system	Induced by TGF-β; cooperates with SMAD proteins to activate TGF-β/BMP target gene transcription and mediate TGF-β-induced growth arrest	Human epithelial cell lines (Mv1Lu, HaCaT, HCT116), mouse C2C12 cell line (osteogenic differentiation model), HEK293T cells	[76,83]

Abbreviations: TF, transcription factor; HSCs, hepatic stellate cells; UUO, unilateral ureter obstruction; EMT, epithelial-to-mesenchymal transition.

**Table 3:** Nominal associations between transcription factor expression changes and EMPA-induced differential allele-specific expression (dASE) of their putative targets in vivo.

Target Gene	SNP	N	Patient IDs	TF	β	95% CI	R^2^	*p*	*p*.adj	*p*.adj Global	LOO β Range	LOO Max *p*
*ADAM10*	rs514049	6	s1, s2, s5, s6, s9, s10	RARA	–3.27	(–4.53, –2.01)	0.93	0.002	0.036	0.596	(–3.53, –2.79)	0.011
*AMPD3*	rs7938316	3	s5, s7, s9	NFKB2	1.28	(1.19, 1.38)	1.00	0.004	0.044	0.596	NA	NA
*BMP8B*	rs804395	5	s1, s3, s4, s8, s9	ZNF888	–3.27	(–4.27, –2.28)	0.97	0.002	0.035	0.596	(–3.48, –3.10)	0.019
*CEP131*	rs9911096	4	s2, s4, s6, s9	IRF6	–0.87	(–1.29, –0.45)	0.98	0.012	0.098	0.839	(–1.39, –0.82)	0.226
*DFFB*	rs4648426	3	s4, s5, s6	ZNF140	–0.66	(–0.93, –0.40)	1.00	0.020	0.040	0.839	NA	NA
*DFFB*	rs6424059	5	s4, s5, s7, s8, s10	MAF	–0.63	(–0.86, –0.41)	0.96	0.003	0.021	0.596	(–0.70, –0.59)	0.029
*DNAJB1*	rs11085895	6	s1, s3, s6, s7, s8, s10	ZNF16	–0.75	(–1.29, –0.20)	0.78	0.019	0.096	0.839	(–0.83, –0.53)	0.103
*EIF2B2*	rs175040	4	s2, s5, s6, s8	KLF9	2.93	(2.58, 3.29)	1.00	0.001	0.013	0.596	(2.86, 2.98)	0.042
*EMP3*	rs8102349	4	s4, s5, s7, s10	TCF4	0.60	(0.43, 0.76)	0.99	0.004	0.060	0.596	(0.57, 0.75)	0.298
*LILRB4*	rs1654668	5	s2, s3, s6, s9, s10	SMAD3	0.35	(0.06, 0.65)	0.83	0.032	0.097	0.839	(0.29, 0.47)	0.268
*MBP*	rs10454721	3	s5, s7, s8	SMAD3	–1.76	(–1.92, –1.59)	1.00	0.005	0.038	0.641	NA	NA
*PFKFB3*	rs11256972	3	s7, s8, s9	KLF16	0.80	(0.75, 0.86)	1.00	0.003	0.038	0.596	NA	NA
*SLC20A1*	rs11123144	3	s4, s6, s10	ATF1	–0.84	(–0.87, –0.81)	1.00	0.002	0.019	0.596	NA	NA
*SULF2*	rs3810526	6	s1, s2, s3, s4, s7, s8	ZNF646	0.47	(0.28, 0.66)	0.93	0.002	0.009	0.596	(0.44, 0.90)	0.010
*TUBG1*	rs9897375	3	s2, s3, s6	ZNF770	13.89	(12.87, 14.91)	1.00	0.004	0.081	0.596	NA	NA
*ZNF737*	rs12979592	4	s2, s5, s7, s10	ZNF449	2.19	(1.06, 3.31)	0.97	0.014	0.084	0.839	(1.85, 2.42)	0.161

Target gene and TF, official HGNC symbols; SNP, rsID of the promoter SNP; N, number of individuals heterozygous for the corresponding SNP; Patient IDs, included individuals; β, regression coefficient describing the association between the change in TF expression (day 1 vs. day 0) and dASE of the target gene; 95% CI, 95% confidence interval of β; R^2^, coefficient of determination; *p*.adj, Benjamini–Hochberg-adjusted *p* value calculated separately for each promoter SNP; *p*.adj global, Benjamini–Hochberg-adjusted *p* value calculated across all tested gene–SNP–TF associations; LOO β range, range of regression coefficients from leave-one-out analysis; LOO max *p*, maximum *p* value obtained across leave-one-out analyses, with *p* > 0.1 indicating failure to retain statistical significance; NA, leave-one-out analysis not performed due to the insufficient sample size (*N* = 3).

## Data Availability

The original contributions presented in this study are included in the article/Supplementary Materials. Further inquiries can be directed to the corresponding author. The code and processed data supporting this study are available at https://github.com/mariagubina/ase_empa_tfs (accessed on 4 August 2026).

## Supplementary Materials

The following supporting information can be downloaded at https://www.mdpi.com/article/10.3390/ijms27167202/s1.

## Abbreviations

The following abbreviations are used in this manuscript:

DM	diabetes mellitus
T2DM	type 2 diabetes mellitus
EMPA	empagliflozin
SGLT2	sodium–glucose cotransporter 2
SGLT2i(s)	sodium–glucose cotransporter 2 inhibitor(s)
PBMCs	peripheral blood mononuclear cells
SNP	single-nucleotide polymorphism
rSNP	regulatory SNP
TF(s)	transcription factor(s)
ASE	allele-specific expression
dASE	differential allele-specific expression
ddASE	inter-individual variability in differential allele-specific expression
