# Supplementary material for: Analysis of Allele-Specific Expression Highlights Novel Participants of Empagliflozin-Driven Effects on T2DM-Associated Regulatory Pathways
Source: Int J Mol Sci. 2026 Aug 12;27(16):7202. doi: 10.3390/ijms27167202 (PMC13513580; doi:10.3390/ijms27167202)
Supplement: Supplementary file 1 [file ijms-27-07202-s001.zip › Supplementary_Figure_S1.pdf]

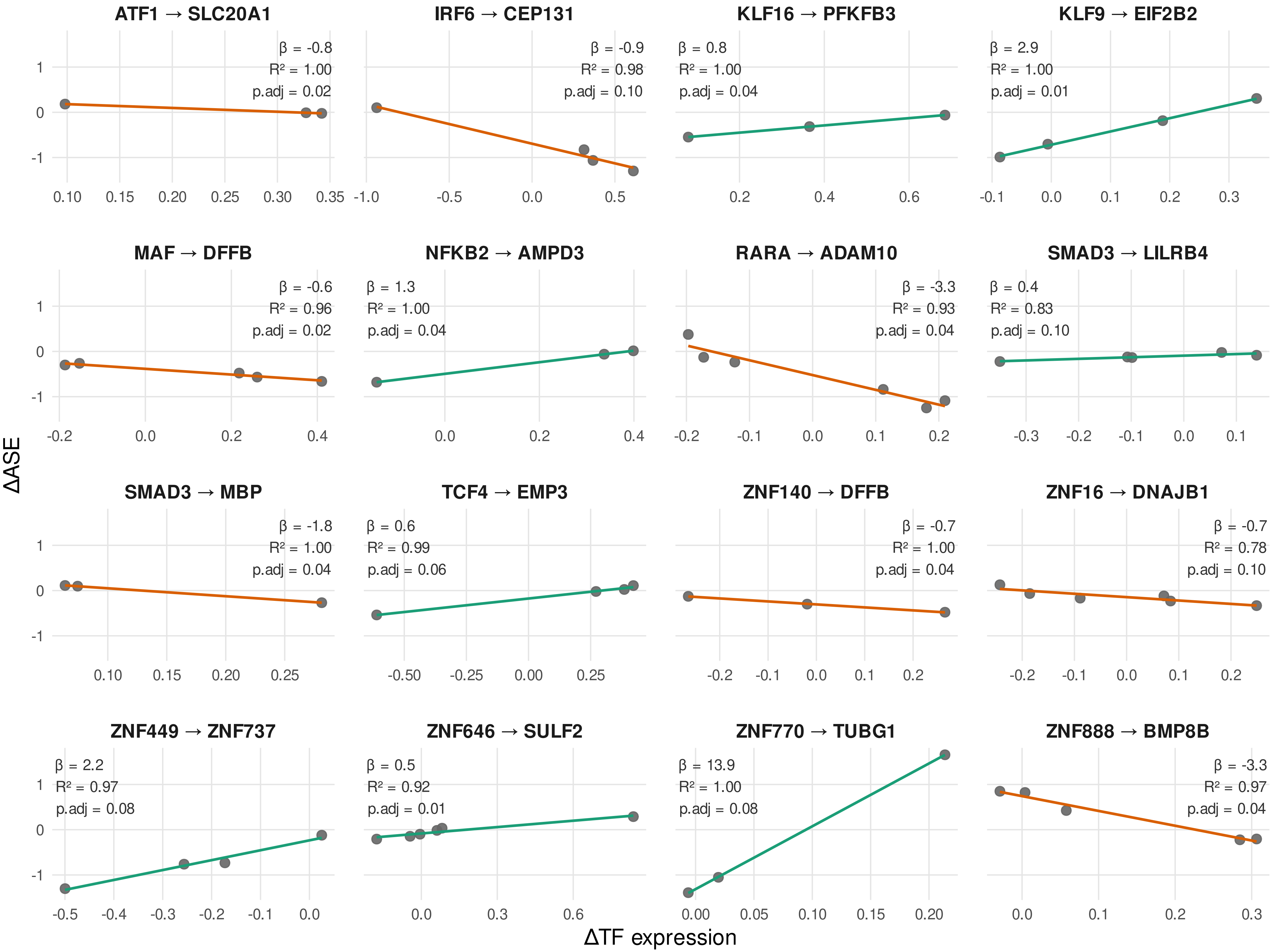

Supplementary Figure S1. Nominal associations between empagliflozin-induced changes in transcription factor expression and differential allele-specific expression of putative target genes in PBMCs from patients with T2DM.

Each panel represents one association identified in the in vivo T2DM cohort (day 1 vs day 0; adjusted *P* < 0.1 after Benjamini–Hochberg correction performed separately for each SNP). Points correspond to individual patients carrying a heterozygous promoter SNP, and the regression line shows the fitted linear model relating dASE to ΔTF expression (dASE ~ $\beta_0 + \beta_1 \times \Delta TF$).
