# Supplementary material for: Analysis of Allele-Specific Expression Highlights Novel Participants of Empagliflozin-Driven Effects on T2DM-Associated Regulatory Pathways
Source: Int J Mol Sci. 2026 Aug 12;27(16):7202. doi: 10.3390/ijms27167202 (PMC13513580; doi:10.3390/ijms27167202)
Supplement: Supplementary file 1 [file ijms-27-07202-s001.zip › Supplementary_Tables S5,S6.pdf]

Supplementary Table S5. Data encoding for anonymization in the clinical trial, experiments and follow-up data processing.

Patient ID within the in vivo cohort	Sex assigned at birth	Age, years
S1	female	56
S2	male	62
S3	male	48
S4	female	65
S5	male	44
S6	female	55
S7	male	58
S8	female	63
S9	female	64
S10	male	65
**Personal ID within the in vitro cohort**	**Sex assigned at birth**	**Age, years**
S101	female	49
S103	female	48
S104	male	32

Patient/Personal ID, the identifier used for subject identification and data encoding; **age**, number of full years at the start of therapy/experiments.

Supplementary Table S6. Clinical characteristics of patients with T2DM at the start of empagliflozin (EMPA) therapy (day 0).

Parameter	Median	Min–max
Age, years	60	44–65
Body mass index, kg/m^2	31.6	27.4–39.9
Waist-to-hip ratio	0.99	0.90–1.13
Duration of diabetes, years	6	2–14
Hemoglobin A1c, %	6.4	5.8–7.9
mmol/mol	46	50–63
eGFR, mL/min×1.73 m^2	89	57–108
Uric acid, μmol/L	310	200–441
Total cholesterol, mmol/L	4.83	3.0–8.31
HDL-cholesterol, mmol/L	1.36	0.93–1.97
LDL-cholesterol, mmol/L	4.53	2.16–6.13
Triglycerides, mmol/L	2.01	0.68–5.56
UACR, mg/mmol	0.52	0.28–8.25
hsCRP, mg/L	2.65	0.72–5.84

eGFR, estimated glomerular filtration rate; **HDL**, high-density lipoprotein; **LDL**, low-density lipoprotein; **UACR**, urinary albumin-to-creatinine ratio; **hsCRP**, high-sensitivity C-reactive protein. Further details on the methodology used to determine these clinical parameters and their changes after therapy are provided in the following publication: Korbut, A.I.; Romanov, V.V.; Klimontov, V.V. Effects of Empagliflozin on Biomarkers of Renal Injury, Fibrosis and Low-Grade Inflammation in Patients with Type 2 Diabetes. *Diabetes mellitus* **2026**, *28*, 550–557, doi:10.14341/DM13350.
